# Redox biology of *Mycobacterium tuberculosis *H37Rv: protein-protein interaction between GlgB and WhiB1 involves exchange of thiol-disulfide

**DOI:** 10.1186/1471-2091-10-1

**Published:** 2009-01-05

**Authors:** Saurabh Garg, Md Suhail Alam, Richa Bajpai, KV Radha Kishan, Pushpa Agrawal

**Affiliations:** 1 Department of Environmental and Biomolecular Systems, OGI School of Science and Engineering, Oregon Health and Science University, 20000 NW Walker Road, Beaverton, Oregon, 97006, USA; 2 Institute of Microbial Technology, CSIR, Sector-39A, Chandigarh, 160036, India; 3 Informatics, GVK Biosciences, Hyderabad, 500037, India

## Abstract

**Background:**

*Mycobacterium tuberculosis*, an intracellular pathogen encounters redox stress throughout its life inside the host. In order to protect itself from the redox onslaughts of host immune system, *M. tuberculosis *appears to have developed accessory thioredoxin-like proteins which are represented by ORFs encoding WhiB-like proteins. We have earlier reported that WhiB1/Rv3219 is a thioredoxin like protein of *M. tuberculosis *and functions as a protein disulfide reductase. Generally thioredoxins have many substrate proteins. The current study aims to identify the substrate protein(s) of *M. tuberculosis *WhiB1.

**Results:**

Using yeast two-hybrid screen, we identified alpha (1,4)-glucan branching enzyme (GlgB) of *M. tuberculosis *as a interaction partner of WhiB1. *In vitro *GST pull down assay confirmed the direct physical interaction between GlgB and WhiB1. Both mass spectrometry data of tryptic digests and *in vitro *labeling of cysteine residues with 4-acetamido-4' maleimidyl-stilbene-2, 2'-disulfonic acid showed that in GlgB, C^95 ^and C^658 ^are free but C^193 ^and C^617 ^form an intra-molecular disulfide bond. WhiB1 has a C^37^XXC^40 ^motif thus a C^40^S mutation renders C^37 ^to exist as a free thiol to form a hetero-disulfide bond with the cysteine residue of substrate protein. A disulfide mediated binary complex formation between GlgB and WhiB1C^40^S was shown by both in-solution protein-protein interaction and thioredoxin affinity chromatography. Finally, transfer of reducing equivalent from WhiB1 to GlgB disulfide was confirmed by 4-acetamido-4' maleimidyl-stilbene-2, 2'-disulfonic acid trapping by the reduced disulfide of GlgB. Two different thioredoxins, TrxB/Rv1471 and TrxC/Rv3914 of *M. tuberculosis *could not perform this reaction suggesting that the reduction of GlgB by WhiB1 is specific.

**Conclusion:**

We conclude that *M. tuberculosis *GlgB has one intra-molecular disulfide bond which is formed between C^193 ^and C^617^. WhiB1, a thioredoxin like protein interacts with GlgB and transfers its electrons to the disulfide thus reduces the intra-molecular disulfide bond of GlgB. For the first time, we report that GlgB is one of the *in vivo *substrate of *M. tuberculosis *WhiB1.

## Background

A large number of cellular processes are mediated through protein-protein interactions. In general, these interactions are non-covalent and are outcome of hydrophobic or ionic or both interactions. In case of thioredoxin (Trx), a protein-protein interaction is followed by the exchange of disulfide from thioredoxin to the substrate protein. Trx is a major protein disulfide reductase responsible for maintaining the redox state of cytosol. They are involved in large number of cell processes and regulate the activity of many proteins through reversible reduction of their disulfide bonds. The specificity of a Trx towards its target is determined by the local environment around its active site [1]. Thioredoxins (Trxs) take electrons for their reduction from NADPH *via *thioredoxin reductase and then transfer them to target disulfide [2]; hence, they play a key role in a cell's defense against oxidative stress. Trxs have a universal folding pattern known as 'thioredoxin fold' and a conserved but solvent exposed CXXC motif, as an active site [3]. In *E*.*coli*, Trxs are also involved in the reduction of disulfide bonds of OxyR, Hsp33 *etc*. and regulate their biochemical function [4]. They often regenerate cellular proteins by reducing non-specific disulfides formed during oxidative stress thus also function as an important antioxidant system. In eukaryotes, Trxs regulate activity of transcription factors NF-κB and AP-1 [5,6]. The activation of peroxiredoxins, which facilitates reduction of reactive oxygen species, is dependent on Trxs [7].

Alpha (1,4)-glucan branching enzyme (GlgB) of *Mycobacterium tuberculosis *(*Mtb*) is encoded by Rv1326c [8]. Earlier, we have reported that the recombinant protein produced by Rv1326c is functionally active [9]. Amino acids sequence analysis showed that both *Mtb *and *M. bovis *GlgB has four cysteine residues (in *Mtb *these are C^95^, C^193^, C^617^and C^658^) but are absent in other mycobacteria. We indicated that cysteine residues of *Mtb *GlgB may form intra-molecular disulfide bond(s) and its reduction leads to a conformational change of the protein [9]. High density transposon mutagenesis [10] showed that *glgB *gene is essential for the optimal growth of *Mtb in vitro*, suggesting that it is an important gene. However, its importance in physiology of *Mtb *has not yet been established. *Mtb *has three different pathways for trehalose biosynthesis [11] where GlgB is likely to be a part of one of the pathways. Glycogen is a mixture of glucose polymers, thus GlgB may help in the synthesis of a precursor for trehalose biosynthesis. Both in mycobacteria and corynebacteria, trehalose is a basic component of a number of cell wall glycolipids [12], cord factor (trehalose 6,6'-dimycolate), sulpholipids (acetylated trehalose-2'-sulphate derivatives) and trehalose containing lipo-oligosaccharides [13]. Lately, enzymes of trehalose biosynthetic pathways have gained major attention as drug targets especially in mycobacteria, as capsular polysaccharides of *Mtb *have been found to modulate the host immune response [14].

In *Streptomyces coelicolor *A3(2), *whiB *gene was shown to be associated with sporulation [15]. *Mtb *does not sporulate but has seven ORFs which are orthologues of *whiB *of *S. coelicolor *[8]. The presence of multiple *whiB *genes in *M. leprae *[16] and the growing body of evidence that these genes are involved in cell division, oxidative stress, antibiotic resistance, pathogenesis *etc. *[17-22], suggests that these genes are likely to have functional significance in mycobacteria. Amino acid sequence analysis of WhiB proteins showed that they all have four conserved cysteines. Except in WhiB5/Rv0022c of *Mtb*, which has a CXXXC motif, all other WhiB proteins have a CXXC motif which is similar to thioredoxins and other oxidoreductases. In mycobacteria, ORFs showing sequence similarities to *whiB *are annotated as putative transcription factors [8]. However, so far the molecular function of WhiB proteins as a DNA binding proteins has not been established. We have reported that WhiB proteins, except WhiB2, have properties similar to thioredoxin [23-26] suggesting that these proteins would function *via *protein-protein interaction. Interaction of thioredoxins with substrate protein involves exchange of thiol-disulfide. However, occasionally thioredoxins also regulate the activity of the cognate protein independent of disulfide exchange [27,28].

In the present study, we show that WhiB1 and GlgB of *Mtb *exchange their thiol disulfide. Initially, GlgB was identified as a possible substrate protein of WhiB1 in a yeast two-hybrid screen. Physical interaction between the two proteins was confirmed by GST pull-down assay. The data obtained from the mass spectrometry and 4-acetamido-4' maleimidyl-stilbene-2, 2'-disulfonic acid (AMS) trapping confirmed that an intra-molecular disulfide bond is formed between C^193 ^and C^617 ^of *Mtb *GlgB. Using a series of biochemical methods; we suggest that GlgB is a substrate of WhiB1.

## Results and discussion

### WhiB1 interacts with GlgB

WhiB1, a thioredoxin like protein is likely to function *via *protein-protein interaction therefore, yeast two-hybrid system was employed to identify the potential interacting partner/s of WhiB1. The suitability of yeast two-hybrid system to study mycobacterial protein-protein interaction has been well demonstrated [21,29].

The expression of *whiB*1 (fused to DNA binding domain of Gal4p, pDBD-B1) was studied by repression assay where the *β*-galactosidase (*β*-gal) activity of *S. cerevisiae *cells transformed with vector pJK101 and pDBD-B1 was measured. In yeast two-hybrid analysis, when bait is expressed and binds to the LexA operator region, the expression of downstream reporter gene (*lacZ *in this study) is repressed. The *β*-gal expression was significantly repressed in the cells transformed with pDBD-B1 whereas, the transformants lacking bait showed increased *β*-gal expression (Additional file 1, Figure 1A). The data clearly suggested that the WhiB1 expressed in the yeast cell and was able to bind to the operator region. The intrinsic transcription activation of bait was ruled out by testing the expression of *β*-gal of *S. cerevisiae *transformed by constructs: pSH18-34 + pJG4-5 + pDBD-B1. Transformants containing: pSH18-34 + pJG4-5 + pSH17-4 and pSH18-34 + pJG4-5+ pRFHM1 were used as positive and negative controls, respectively. Absence of blue color in the test transformants clones (Additional file 1, Figure 1B, panel 1) similar to the negative control clones (Additional file 1, Figure 1B, panel 3) confirmed that WhiB1 lack intrinsic transcription activation property and was suitable to be used in yeast two-hybrid analysis.

**Figure 1:**
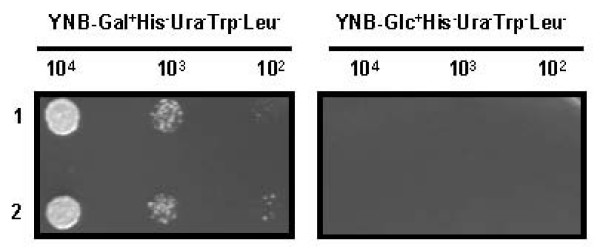
**Yeast two-hybrid analysis using *whiB*1 as 'bait'**. *Saccharomyces cerevisiae *cells were transformed with plasmids: pDBD-B1 (bait), pAD*Mtb *(prey) and pSH18-34 (reporter plasmid) and the transformants were selected on Glu^+^His^-^Ura^-^Trp^- ^plates. The transformants were pooled and allowed to grow in a liquid medium supplemented with galactose (1%) for 6 h at 30°C in order to induce the expression of gene cloned in pAD*Mtb*. After induction, the cells were plated on Gal^+^His^-^Ura^-^Trp^- ^Leu^- ^plates. Viable clones were further tested for specific interaction by dilution plating on Gal^+^His^-^Ura^-^Trp^-^Leu^- ^and Glu^+^His^-^Ura^-^Trp^-^Leu^- ^plates. Growth of two representative positive clones in dilution plating is shown in the picture. Figures on the top indicate the total number of cells spotted on the respective plate for dilution plating.

To search for an interaction partner, *S. cerevisiae *cells were transformed with pDBD-B1 (bait) and the genomic library of *Mtb *H37Rv, which was created as a fusion to the activation domain (pAD*Mtb*) of Gal4P. The system used in this study is based upon the transcription of a reporter gene which occurs when the two separately expressed domains of Gal4p physically interact. Therefore, when the two proteins are expressed as a fusion to two different domains of Gal4p, their physical interaction leads to functional reconstitution of Gal4p thereby leading to the activation of expression of reporter genes, *LEU*2 or *lacZ*, when grown in a medium containing galactose but not glucose. At the end of the screening process, we obtained sixteen clones exclusively growing on galactose medium in the absence of leucine (Figure. 1). Among all, 15 clones had an in-frame fusion with 596 bp 3' end of ORF Rv1326c/*glgB *whereas one was false positive (vector sequence alone).

**Figure 2:**
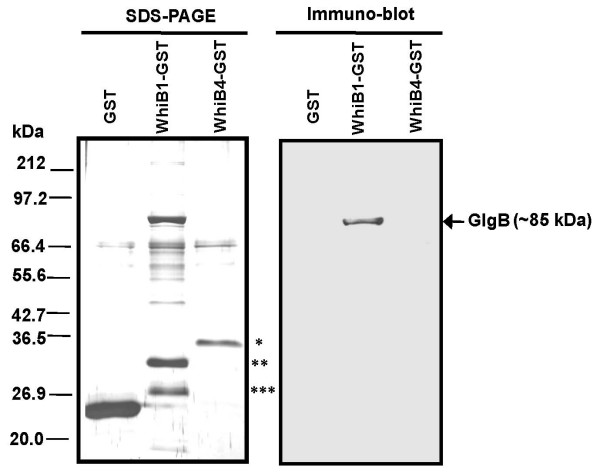
**WhiB1 physically interacts with GlgB *in vitro***. GST pull down assay to show GlgB interacts with WhiB1. WhiB1-GST/WhiB4-GST/GST-alone were co-expressed with GlgB-6× His in *E. coli *BL21(DE3). Interaction was carried out using glutathione agarose beads as described in 'materials and methods'. The left panel is 10% SDS-PAGE stained with silver nitrate, whereas the right panel is an immuno-blot showing the co-elution of GlgB along with WhiB1-GST. The asterisks denote the position of WhiB4-GST (*), WhiB1-GST (**) and GST-alone (***).

Results obtained by yeast-two hybrid analysis were further confirmed by GST pull-down assay. WhiB1 and WhiB4 proteins were expressed as a fusion to GST (pGEX-B1 and pGEX-B4). Both, pGEX-B1 and pGEX-B4 (control) were co-expressed separately with pETgl (full length *glgB *cloned in pET29a to express it as a C-terminus 6-× His tagged fusion protein). The proteins were immobilized on glutathione sepharose resin. The absence of GlgB in lanes: GST alone and WhiB4-GST (Figure. 2) suggested that the interaction between WhiB1 and GlgB is specific. It is evident from the Figure. 2 that WhiB1 interacts with GlgB but not with WhiB4 or GST alone, suggesting that the interaction of GlgB and WhiB1 is specific. In the pull down assay a ~27 kDa protein was co-eluted. N-terminal amino acids sequencing of this protein identified it as a cleaved GST.

The interaction data was surprising because to the best of our knowledge, interaction of a Trx-like protein with an enzyme of sugar metabolic pathway has not been reported in any microorganism except cyanobacteria. Amino acids sequence analysis of GlgBs from different organisms showed that except *Mtb *and *M. bovis*, no other GlgB has four cysteine residues. Earlier, we have reported that *Mtb *GlgB has at least one intra-molecular disulfide bond [9]. Therefore, we investigated the number and arrangement of intra-molecular disulfide bond(s) in GlgB.

### C^193 ^and C^617 ^of GlgB form an intra-molecular disulfide bond

**Figure 3:**
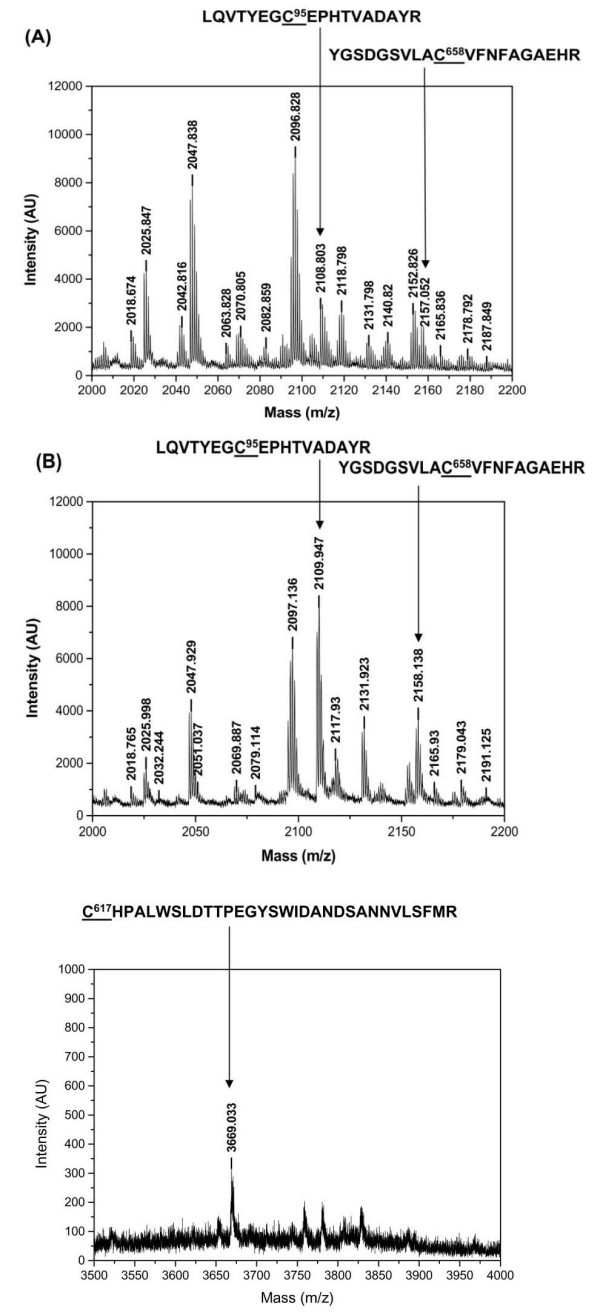
**Peptide mass fingerprinting of GlgB**. DTT treated and untreated GlgB was alkylated, digested with trypsin and precipitated with TCA. The molecular mass of the peptides were analyzed by MALDI-TOF. (A) Peptide mass spectra highlighting the molecular mass of peptide containing C^95 ^and C^658 ^(marked with arrow) under oxidizing conditions. (B) Peptide mass spectra under reducing conditions. The molecular mass of the peptides containing cysteines (as labeled) were similar to the peptide obtained under oxidized conditions, confirming that both C^95 ^and C^658 ^are free and surface exposed. (C) Peptide mass spectra of GlgB pre-incubated at 45°C before reduction, alkylation and trypsinization. Peptide containing C^617 ^is marked with an arrow; however C^193 ^could not be detected.

The peptide mass finger printing analysis of tryptic digests of cysteine alkylated and non alkylated GlgB showed that both C^95 ^(LQVTVEG**C**EPHTVADAYR) and C^658 ^(VGSDGSVLA**C**VFNFAGAEHR) were alkylated under oxidizing conditions suggesting that these two cysteine residues were free and surface exposed (Figure. 3A &3B), while peptides containing either C^193 ^or C^617 ^could not be detected. Surprisingly, we could not detect the peptide containing C^193 ^and C^617 ^even under normal reducing conditions. However, incubation of reduced GlgB at 45°C followed by alkylation and trypsin digestion resulted in to the detection of peptide (**C**HPALWSLDTTPEGYSWIDANDSANNVLSFMR) containing C^617^(Figure. 3C) but not the peptide (VLGPSGVWELFWPDFP**C**DGLYK) containing C^193^. The result indicated that under normal reducing conditions, the peptide with C^193 ^and C^617 ^might be buried and was not accessible to trypsin, whereas at 45°C, possibly a change in conformation led to the exposure of an internal trypsin recognition site which released the peptide with C^617 ^but not the peptide stretch containing C^193^. Based upon our earlier report [9] and the present mass spectrometry data, we propose that an intra-molecular disulfide bond is formed between C^193 ^and C^617^.

**Figure 4:**
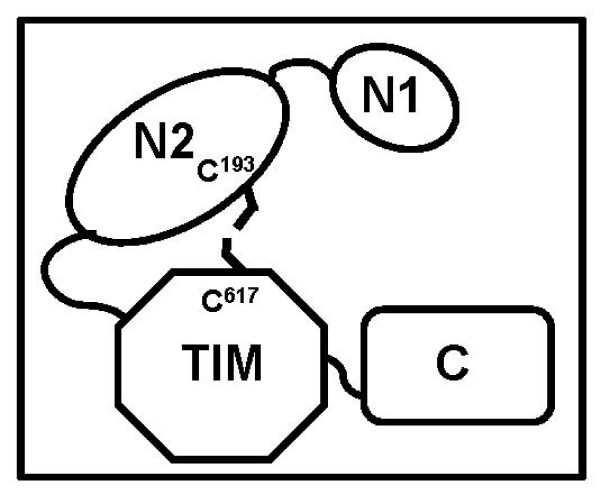
**Possible domain organization of *Mtb *GlgB**. The schematic arrangement of the four domains of *Mtb *GlgB is based on mass spectrometric data and structure model. Cysteines (C^193 ^and C^617^) of GlgB which form an intra-molecular disulfide bond are indicated.

In order to know about the domain organization in *Mtb *GlgB, a structure model was generated (Additional file 1, Figure 2) using the structure of *E. coli *GlgB as a template (the proteins shares 48% sequence identity and 68% similarity). The *E. coli *GlgB structure was determined without one of the N-terminal domains (N1) because of crystallization related problems [30-32]. The *E. coli *GlgB contains three laterally arranged domains *i.e*. N2, catalytic (TIM-barrel) and the C-terminal domains. The relative orientation and function of the N1 domain is not yet known and it is assumed that it possibly regulates the size of sugar branching during glycogen synthesis [33]. The model indicated that *Mtb *GlgB is also likely to have four domains. Interestingly, each of the domains of *Mtb *GlgB has one cysteine residue (C^95 ^in N1, C^193 ^in N2, C^617 ^in active site and C^658 ^in C-terminus). It appears that an intra-molecular disulfide bond may link the N2 and catalytic domains and after reduction, the two domains would have separated which exposed the C^617 ^while the N2 domain, especially the region containing C^193 ^might have got re-oriented therefore was not accessible for trypsinization. This could be a possible reason why the peptide containing C^193 ^was not detected in mass spectrometry. Based upon the mass spectrometry data and structural model, a possible scheme of domains organization in oxidized GlgB has been depicted in the Figure 4.

### WhiB1 and GlgB interaction involves exchange of thiol-disulfide

**Figure 5:**
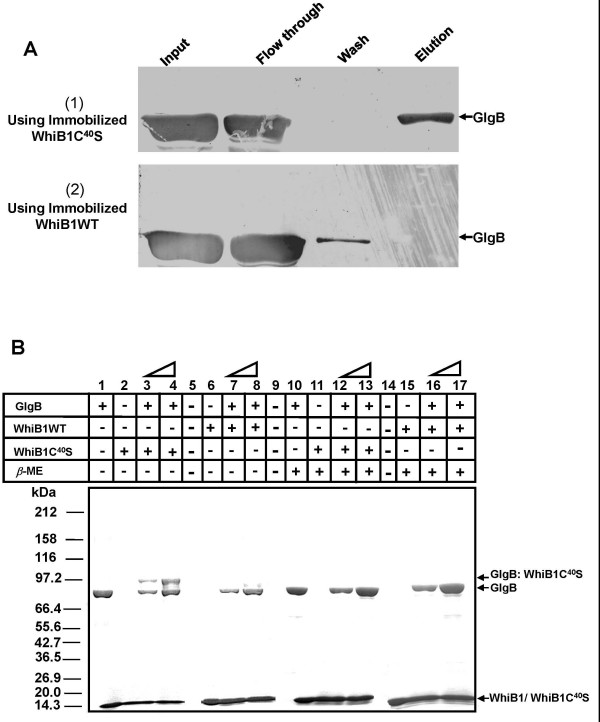
**Thioredoxin affinity chromatography**. (A) Immuno-blot after thioredoxin affinity chromatography to show that the binary complex between GlgB and WhiB1C^40^S was destabilized by DTT. Panels: (1) interaction of GlgB with WhiB1C^40^S, (2) interaction of GlgB with WhiB1WT. (B) In-solution protein-protein interaction to show the formation of a binary complex between GlgB and WhiB1C^40^S. After reactions, the samples were resolved by 8–18% step gradient SDS-PAGE and stained with coomassie blue. A binary complex between WhiB1C^40^S and GlgB at ~97 kDa marker position (lanes 3 & 4) disappeared when *β*-ME was added (lanes 12 & 13). Rests of the lanes are controls as labeled. The triangles represent the interaction carried out using two different concentrations of WhiB1 and GlgB (5 or 10 μM of each protein).

To prove the hypothesis that interaction of WhiB1 with GlgB may involve exchange of thiol-disulfide, the Trx affinity chromatography method, originally developed by Motohashi *et al. *(2001) [34] was employed. First of all, a WhiB1C^40^S mutant was generated, which rendered C^37 ^to remain as a thiolate ion. To demonstrate the formation of hetero-disulfide bond between GlgB and WhiB1, purified WhiB1C^40^S or WhiB1WT (control) proteins were covalently immobilized on CNBr activated sepharose resin. Both C^95 ^and C^658 ^of GlgB were alkylated under oxidizing condition to prevent a non-specific reaction between free thiols of GlgB and C^37 ^of WhiB1. Interaction was initiated by mixing immobilized WhiB1C^40^S and pre-alkylated oxidized GlgB. The reaction was terminated at different time points and samples were resolved by non-reducing PAGE. The Figure 5A clearly shows that GlgB formed a complex with WhiB1C^40^S just after 10 minutes of interaction unlike WhiB1WT where GlgB was washed out completely in high salt buffer. The result indicated that a binary complex formed between GlgB and WhiB1C^40^S was stabilized by a hetero-disulfide bond.

In Trx affinity chromatography, the binary complex formed between the two proteins is not released because WhiB1/WhiB1C^40^S is covalently bound to the resin. In order to demonstrate that GlgB and WhiB1 are indeed linked by a hetero-disulfide bond, purified pre-alkylated oxidized GlgB and WhiB1C^40^S were incubated together in the interaction buffer. The reaction was terminated at different time points and the samples were resolved by SDS-PAGE. A higher molecular weight protein band (~97 kDa) along with the two input bands of GlgB (~85 kDa) and WhiB1C^40^S (~14 kDa), was clearly visible (Figure. 5B, lanes 3 & 4). The ~97 kDa protein band corresponded to the combined sizes of GlgB: WhiB1C^40^S binary complex which disappeared when 1% *β*-ME was added to the sample (Figure. 5B, lanes 12 & 13), thus confirming the formation of an inter-molecular hetero-disulfide bond between GlgB and WhiB1C^40^S. As expected, the ~97 kDa band was missing in the GlgB: WhiB1WT control sample (Figure. 5B, lanes 7 & 8) because the intact CXXC motif of WhiB1WT could not form a stable inter-molecular hetero-disulfide bond with GlgB.

**Figure 6:**
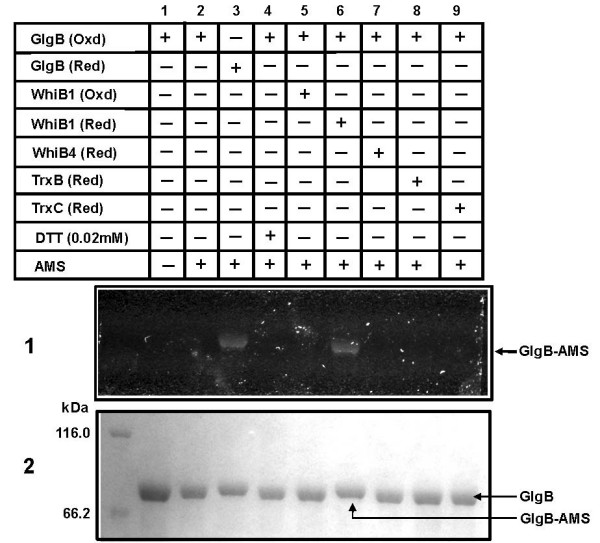
**Reduction of GlgB's disulfide by reduced WhiB1**. After interaction between WhiB1 and GlgB, the reduced cysteines of GlgB were trapped using AMS and the samples were resolved by 8% SDS-PAGE. Panels are: (1) resolved GlgB on gel, as visualized under UV light, the fluorescent band shows the binding of AMS to reduced cysteines of GlgB as labeled, (2) coomassie stained gel (after photographed under UV light) to show the presence of GlgB. The AMS labeled GlgB also showed mobility shift. Lanes are indicated on the top.

The direct evidence for the reduction of GlgB disulfide by WhiB1 was shown using AMS trapping method. The fluorescent dye, AMS, absorbs light at 322 nm and emits at 411 nm. Binding of each molecule of AMS also increases the mass of a protein by ~500 Da. Thus, AMS labeled proteins fluoresce under UV light and show reduced mobility on SDS-PAGE. Purified *Mtb *GlgB was pre-alkylated under oxidizing condition with iodoacetamide (IAA) to block the free cysteines thus AMS binding would occur if C^193 ^and C^617 ^are free either due to the reduction by WhiB1 or DTT or by other Trx-like proteins. The AMS treatment of oxidized and pre-alkylated GlgB did not show any fluorescence or mobility shift (Figure. 6, lane 2), suggesting that the free cysteines were successfully alkylated. Pre-alkylated GlgB when reduced by DTT and then labeled with AMS fluoresced under UV light and also showed mobility shift (Figure. 6, lane 3). Similarly, when pre-alkylated GlgB was incubated with pre-reduced WhiB1 and labeled with AMS, it fluoresced under UV light and showed mobility shift on the gel (Figure. 6, lane 6). However, pre-alkylated GlgB incubated with oxidized WhiB1 and then labeled with AMS failed to fluoresce and also did not show any mobility shift (Figure. 6, lane 5). Together, these data suggested that WhiB1 converted the disulfide of GlgB into reduced thiol, to which AMS reacted covalently. In order to know whether GlgB's disulfide could be reduced by other Trxs of *Mtb*; TrxB and TrxC of *Mtb *were cloned, expressed and proteins were purified (Additional file 1, Figure 3). Both, TrxB and TrxC catalyzed the reduction of insulin disulfides by DTT suggesting that the proteins were in native conformation and enzymatically active (Additional file 2, Table 1). However, incubation of GlgB with either reduced WhiB4 or TrxB or TrxC did not show AMS labeling of GlgB (Figure. 6, lane 7–9). The data clearly suggested that the transfer of reducing equivalents from WhiB1 to GlgB is a highly specific reaction, as its absence could not be compensated either by known Trxs or WhiB4 of *Mtb*. The result also confirmed that C^193 ^and C^617 ^of GlgB form an intra-molecular disulfide bond.

### WhiB1 promoter activates at late stationary phase

**Figure 7:**
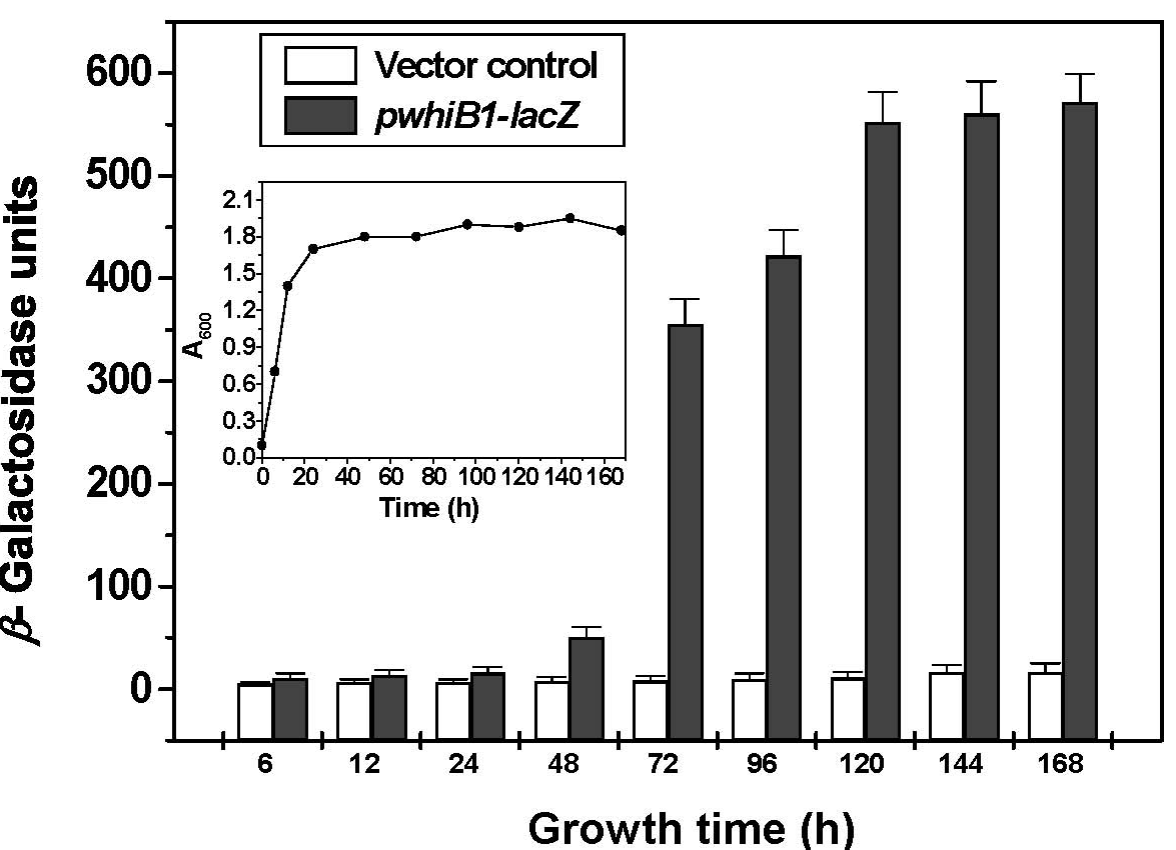
**Growth phase dependent activation of putative *Mtb whiB*1 promoter in *M. smegmatis *mc2155**. *M. smegmatis *cells carrying pJEM15 (vector control, white bar) or *pwhiB*1*-lacZ *(grey bar) were aerobically grown for different periods as indicated. Cells were aliquoted at different time points (indicated as h, at the bottom of each bar) and *A*_600 _was measured followed by the preparation of whole cell lysate. The *β*-gal activity was measured in standard 'Z' buffer. *Inset*: Growth curve of *M. smegmatis *cells.

For a successful interaction between two proteins, it is essential that both are present in the cytosol at the same time. The glycogen synthesis in *S. coelicolor *A3(2) has been shown to be associated with sporulation which represent stationary phase of the growth [35]. In order to determine the stage when *whiB*1 is expressed, the upstream regulatory sequence of *whiB*1 was cloned upstream to the *lacZ *of a promoter less vector pJEM15 [36]. The expression of *β*-gal was monitored at different stages of growth. For the first 24 h, the expression was at the basal level. However, it induced after 48 h of growth and increased suddenly by 72 h (Figure. 7). The increase in *β*-gal activity continued up to 120 h and saturated afterwards, suggesting that the *whiB*1 expression is likely to be at the late stationary phase of the growth. Increased level of *whiB*1 transcripts in stationary phase has also been demonstrated by RT-PCR [19]. Therefore, it is possible that interaction between GlgB and WhiB1 is a stationary phase phenomenon.

Interaction of GlgB with Trxs has been reported in chloroplast and *Synechocystis sps. *[37]. Although, a Trx-mediated post-translational regulation of GlgB in the photosynthetic organisms has been indicated but the biochemical evidence to this has not been demonstrated yet. Earlier, we have shown a redox mediated conformational change in *Mtb *GlgB [9]. Therefore, it is possible that under oxidizing conditions (*in vivo *oxidative stress); an intra-molecular disulfide bond formed between C^193 ^and C^617^. This would possibly bring a conformational change in GlgB which may introduce steric constrain thereby can affect the stability of the protein. Recently, WhiB1 has been demonstrated to co-ordinate an oxidation labile iron-sulfur cluster [26]. It appears that under oxidative stress and/or stationary phase (mild oxidative stress), WhiB1 transforms from inactive holo-form to active apo-form, interacts with GlgB and reduces its oxidized disulfide. WhiB1-mediated reduction of oxidized disulfide would possibly regenerate the GlgB by bringing back to sterically stable state. Therefore, the presence of sterically stable GlgB in stationary phase of the growth will be beneficial to cell where metabolism is usually very low thus, bacteria would avoid any excess consumption of ATP. However, at this juncture due to the lack of experimental evidence, it will be pre-mature to pinpoint the importance of GlgB: WhiB1 interaction and exchange of thiol-disulfide under *in vivo *conditions. Nevertheless, the results described in this study as well as the methods used, would certainly help in deducing many thiol mediated regulatory processes in *Mtb*.

## Conclusion

The data described here show for the first time that GlgB, an enzyme of carbohydrate metabolism is a substrate of WhiB1, a Trx-like protein of *Mtb*. Since, the disulfide bond formation so far, appears to be unique to *Mtb *and may be to *M. bovis *also; both GlgB and WhiB1 are likely to have much more important role in these two species of mycobacteria than others. Although, in yeast-two hybrid analysis we found only GlgB as an interacting protein of WhiB1 however, it does not mean that WhiB1 will not interact with any other protein. Therefore, a detail analysis of protein-protein interaction between WhiB1 with other proteins of *Mtb *is required to understand entire network, which may exist during stationary phase. Further, it is also possible that interaction between WhiB1 and some other protein does not involve thiol-disulfide exchange because Trxs from other systems have been shown to act as structural components of other protein [38,39].

## Methods

### Bacterial strains and reagents

*Escherichia coli *DH5α was used for general cloning procedures. Media components were purchase from Difco, USA. T4 DNA ligase was purchased from Promega (USA) while other restriction and DNA modifying enzymes were procured from New England Biolabs (USA). The primers were obtained from Biobasic, Inc. (Canada). Plasmid pET-29a and *E. coli *BL21(DE3) were purchased from Novagen (USA). Ni^2+^-NTA agarose resin and other PCR/plasmid purification kits were supplied by Qiagen (Germany). The EDTA free protease inhibitor cocktail was purchased from Roche, (Germany). All other fine chemicals and antibodies were procured either from Sigma (USA) or otherwise mentioned. Standard recombinant DNA techniques were followed as described elsewhere [40].

### Cloning, expression and purification of recombinant proteins

Genomic DNA of *Mtb *was isolated using a method described earlier [41]. Cysteine to serine mutation of WhiB1 was generated by overlapping extension PCR using a primer pair consisting of wild type [23] and appropriately mutated primer as mentioned in the Table 2 (Additional file 2). Cloning, expression and purification procedures of mutant proteins were as described for WhiB1WT [23]. Over-expression and purification of GlgB was carried out as described earlier [9] whereas, the expression and purification method of WhiB4 has been described elsewhere [25]. Both *trx*B/Rv1471 and *trx*C/Rv3914 of *Mtb *were PCR amplified from the genomic DNA using sequence specific primers (Additional file 2, Table 2) and cloned into vector pET-29a at *Eco*RI-*Xho*I and *Bam*HI-*Hind*III sites, respectively. Proteins were over-expressed at 30°C by 0.5 mM IPTG for 3 h and purified using Ni^2+^-NTA affinity chromatography following manufacturer's instructions (Qiagen, Germany).

### Yeast two-hybrid assay

*Saccharomyces cerevisiae *EGY191 was used throughout in yeast two-hybrid analysis. 'Bait' and 'prey' fusions were constructed in pEG202 and pJG4-5, respectively as suggested by Golemis and Brent (1997) [42]. *whiB*1/Rv3219 was PCR amplified using gene specific primers (Additional file 2, Table 2) and *Mtb *genomic DNA as template. The gene was cloned at *Eco*RI-*Xho*I site of pEG202 to create pDBD-B1 (bait). Characterization of 'bait': (a) expression in yeast and (b) lack of intrinsic transcriptional activity, was carried out using the methods described elsewhere [42]. The *Eco*RI-*Xho*I digested genomic DNA of *Mtb *were cloned at the same sites of pJG4-5 to express *Mtb *genomic library in fusion with the activation domain of acid blob under *gal *promoter (pAD*Mtb*) and was transformed into *S. cerevisiae*, along with the standard control vectors [42]. The transformants were selected on respective drop out plates and tested for the activation of reporter genes *i.e. LEU2 *and *lacZ*. The selected clones were sequenced in an automated DNA sequencer (ABI 310) and analyzed by the 'Blastn' server (NCBI, NIH).

### GST pull down assay

The PCR amplified fragments of *whiB*1 and *whiB*4/Rv3681c were cloned at *EcoR*I-*Xho*I sites of vector pGEX4T-1 to generate fusion plasmids pGEX-B1 and pGEX-B4, respectively. Both WhiB1 and WhiB4 (internal control) were expressed as N-terminus GST fusion proteins. The pET-gl and pGEX-B1 or pGEX-B4 was co-transformed in *E. coli *BL21 (DE3) cells and protein expression was induced by 0.5 mM IPTG for 3 h at 30°C. The harvested cells were re-suspended in 1 ml lysis buffer: 50 mM Tris.HCl, pH 8.0, 100 mM NaCl, 1 mM EDTA containing protease inhibitors cocktail. After one freeze-thaw cycle, lysozyme (2 mg/ml) was added, incubated for 1 h at 37°C and sonicated. The soluble cell lysate was separated by centrifugation at 17,000 g for 30 minutes. The crude lysate of co-expressing test proteins (500 μg) was mixed with 3 mg of pre-equilibrated glutathione agarose beads and the reaction was carried out in the pull-down buffer: 100 mM Tris.HCl, pH 8.0, 150 mM NaCl, 1 mM EDTA, 0.1% Triton X-100, and 1 mM DTT, at 4°C for 3 h with continuous mixing. The beads were washed five times with 1 ml of pull-down buffer then mixed with gel loading buffer, boiled and the bound proteins were resolved on 10% SDS-PAGE. The gel was stained with silver nitrate [40]. Co-elution of GlgB and WhiB1 was confirmed by immuno-blot using anti-6×His tag monoclonal antibodies.

### Mass spectrometry

To investigate the presence of disulfide bond(s) in GlgB, 100 μg purified protein was incubated with 10 mM DTT for 1 h at 25°C. Both DTT treated and untreated proteins were incubated for 12 h with 20 mM IAA at 37°C in dark and then digested with 2 μg of proteomics grade trypsin for 10 h at 25°C. The digested proteins were precipitated by 10% trichloroacetic acid (TCA) for 15 minutes in ice, washed twice with chilled acetone, air dried and re-suspended in 0.1% TFA. Two μl of each protein sample was mixed with an equal volume of the matrix and mass spectra were recorded in Brukers, Ultraflex MALDI-TOF spectrometer. In another set of experiments, GlgB was incubated at 45°C for 4 h before DTT treatment, alkylated with IAA, digested with trypsin, precipitated by TCA and mass spectra were recorded.

### Trx affinity chromatography

The Trx affinity chromatography method of Motohashi *et al *[34] was used to study the thiol-disulfide exchange between GlgB and WhiB1. WhiB1C^40^S (5 mg) was immobilized on cyanogen bromide (CNBr) activated sepharose beads and was equilibrated with 5 column volumes of binding buffer: 50 mM Tris.HCl, pH 8.0, 100 mM NaCl, 0.05% Triton X-100, 2 mM EDTA, 10 mM MgCl_2_. To the equilibrated resin, 1 mg GlgB (oxidized and pre-alkylated with IAA was mixed and interaction was carried out at 25°C in the presence of protease inhibitor cocktail. Samples were removed every 10 minutes and washed with buffer: 50 mM Tris.HCl, pH 8.0, 0.5 M NaCl until the wash was free of any protein. The bound protein was eluted in 3 ml wash buffer supplemented with 10 mM DTT, precipitated by TCA and resolved by 10% SDS-PAGE. Immuno-blotting was carried out using monoclonal anti-6× His tag antibodies.

### Demonstration of hetero-disulfide linked binary complex formation by SDS-PAGE

GlgB and WhiB1C^40^S or WhiB1WT were mixed in equimolar concentrations (5 or 10 μM) in a buffer: 50 mM Tris.HCl, pH 8.0, 100 mM NaCl, 0.05% Triton X-100, 2 mM EDTA and 10 mM MgCl_2 _for 2 h at 25°C. After reaction, gel loading buffer with or without *β*-mercaptoethanol (β-ME) was added to the samples, resolved by 8–18% step gradient SDS-PAGE and were visualized by coomassie staining.

### AMS trapping by reduced thiol of GlgB

WhiB1, WhiB4, TrxB and TrxC were reduced with 0.5 mM DTT in 50 mM Tris.HCl, pH 8.0, 100 mM NaCl for 1 h at 25°C. Transfer of reducing equivalents from reduced WhiB1 or other proteins (10 μM) to GlgB (5 μM) was studied by incubating them together in a reaction buffer: 50 mM Tris.HCl, pH 8.0, 100 mM NaCl, 0.05% Triton X-100, 2 mM EDTA, 10 mM MgCl_2 _for 10 minutes at 25°C in a 50 μl reaction volume. The concentration of reduced WhiB1/other proteins were adjusted to achieve a final concentration of DTT to 0.02 mM in the reaction mixture because 0.02 mM DTT was not enough to reduce the amount of GlgB used in the present study. Oxidized GlgB and WhiB1 were incubated together with or without 0.02 mM DTT and were used as controls. WhiB4, TrxB and TrxC were also used as internal controls to test the specificity of interaction between GlgB and WhiB1. Reactions were stopped by adding 10% TCA. The proteins were precipitated, air dried, re-suspended in 10 μl 15 mM AMS (Molecular Probes, USA) in a buffer: 6 M urea, 200 mM Tris.HCl, pH 9.0, 10 mM EDTA, 0.5% SDS (w/v) and were incubated at 37°C for 1 h in dark. Labeled proteins were resolved in 8% non-reducing SDS-PAGE and visualized under UV light followed by coomassie staining.

### Promoter activation assay

A 279 bp region between *whiB*1/Rv3219 start codon and the end of Rv3218 was cloned into the blunt ended site of *Sac*II (upstream to the *lacZ *gene) of the vector pJEM15. The recombinant construct was named *pwhiB1-lacZ*. After confirming the sequence and orientation of the cloned fragment, it was transformed into *M. smegmatis *mc^2^155. Vector alone was used as a control. Three independent clones were inoculated into 20 ml Middlebrook 7H9 medium supplemented with OADC for 24 h (*A*_600 _= ~1.8). From this, a fresh medium was inoculated (1%) and grown at 37°C, cells were collected at different time points: 2, 3, 4, 5 and 6 days of growth and lysates were prepared. An equal amount of protein in triplicate was assayed for *β*-gal activity using Miller's method [43]. The assay was set in a 96 well plate in a 100 μl reaction volume which had 1 mg/ml protein and 0.8 mg/ml of ONPG dissolved in 'Z buffer': 60 mM Na_2_HPO4, 40 mM NaH_2_PO4, (pH 7.0), 10 mM KCl, 1 mM MgSO4 and 2.7 μl/ml *β*-ME. As yellow color started to appear, the reaction was stopped by adding 50 μl of 1 M Na_2_CO_3 _and *A*_420 _was measured. The *β*-gal activity was calculated in Miller's units by using the formula *A*_420_/protein concentration (mg/ml) × time in minutes.

### Miscellaneous

WhiB1 concentration was estimated using molar extinction coefficient (∈_280 nm _= 17550 M^-1^cm^-1^). Protein concentration of cell lysate and GlgB was estimated by Bradford's method using BSA as a standard. The nucleotide sequence of all recombinant plasmids were authenticated by sequencing both strands of DNA in an ABI 321 automated DNA sequencer.

Except in GST pull down assay, throughout the interaction studies, pre-alkylated GlgB was used. Briefly, free cysteines (C^95 ^and C^658^) of GlgB were blocked by incubating the protein in 50 mM Tris.HCl, pH 8.0, 100 mM NaCl and 50 mM IAA for 12 h at 37°C in dark. The unbound IAA was removed by extensive dialysis against buffer: 50 mM Tris.HCl, pH 8.0, 100 mM NaCl.

## Abbreviations

AMS: 4-acetamido-4' maleimidyl-stilbene-2: 2'-disulfonic acid; GlgB: α-(1,4)-glucan branching enzyme; *Mtb*: *Mycobacterium tuberculosis*; IAA: iodoacetamide; Trx: thioredoxin; GST: glutathione S-transferase; CNBr: cyanogen bromide; DTT: dithiothreitol; *β*-ME: *β *mercaptoethanol

## Authors' contributions

SKG and MSA carried out all the expression; purification; mutagenesis and biochemical analysis together. KVRK carried out the molecular modeling of *Mtb *GlgB. RB helped in carrying out some of the biochemical studies. SKG, MSA and PA designed the experiments. MSA and PA together wrote the manuscript. PA oversaw the study. All authors read and approved the final manuscript.

## Supplementary Material

Additional file 1 **Figure 1 – Characterization of WhiB1 as a bait**. (A) Repression assay to asses the expression and binding of 'bait' to the LexA operator. Four independent transformants of each of the clones were assayed for *β*-gal expression. *Saccharomyces cerevisiae *cells (~2 × 10^7^) containing constructs (labeled on the x-axis), were permeabilized with 3 drops of chloroform and 2 drops of 0.1% SDS by vigorous vortexing. Reaction was initiated by adding 0.2 ml ONPG (4 mg/ml) in 'Z buffer' for 3.5 minutes at 28°C. The *β*-gal activity is expressed as units, on y-axis. (B) WhiB1 lacks intrinsic transcription activity. *Saccharomyces cerevisiae *cells were transformed with pDBD-B1 (bait), pSH18-34 which contains *β*-gal as a reporter gene and library plasmid pJG4-5. The *β*-gal expression was monitored on the Gal^+^Raff^+^Ura^-^X-gal plate. The panels indicate *S. cerevisiae *containing (1) pSH18-34 + pJG4-5 + pDBD-B1, test (2) pSH18-34 + pJG4-5+ pSH17-4, positive control (3) pSH18-34 + pJG4-5 + pRFHM1, negative control. Figure 2 – Homology model of *Mtb *GlgB. Three domains are colored differently: N2 domain-orange; TIM-barrel domain-magenta; C-terminal domain-blue. The linker regions between the domains are shown in red. The position of the cysteine residues (C^193^, C^617^and C^658^) on the individual domains is shown as green sticks encircled in dotted boundaries. Figure 3 – Over-expression and purification of TrxB/Rv1471 (1) and TrxC/3914 (2) of *Mtb*. The proteins were overexpressed in *E. coli *BL21 (DE3) and were purified by Ni^2+^-NTA affinity chromatography. Lanes are indicated on the top. Click here for file

Additional file 2 **Table 1 – Protein disulfide reductase activity of *Mtb *TrxB and TrxC**. Table 2 – List of primers used in this study Click here for file
