# Supplementary figures and images for: Redox biology of Mycobacterium tuberculosis H37Rv: protein-protein interaction between GlgB and WhiB1 involves exchange of thiol-disulfide
Source: BMC Biochem. 2009 Jan 5;10:1. doi: 10.1186/1471-2091-10-1 (PMC2631452; doi:10.1186/1471-2091-10-1)

## Additional file 1

Figure. 1

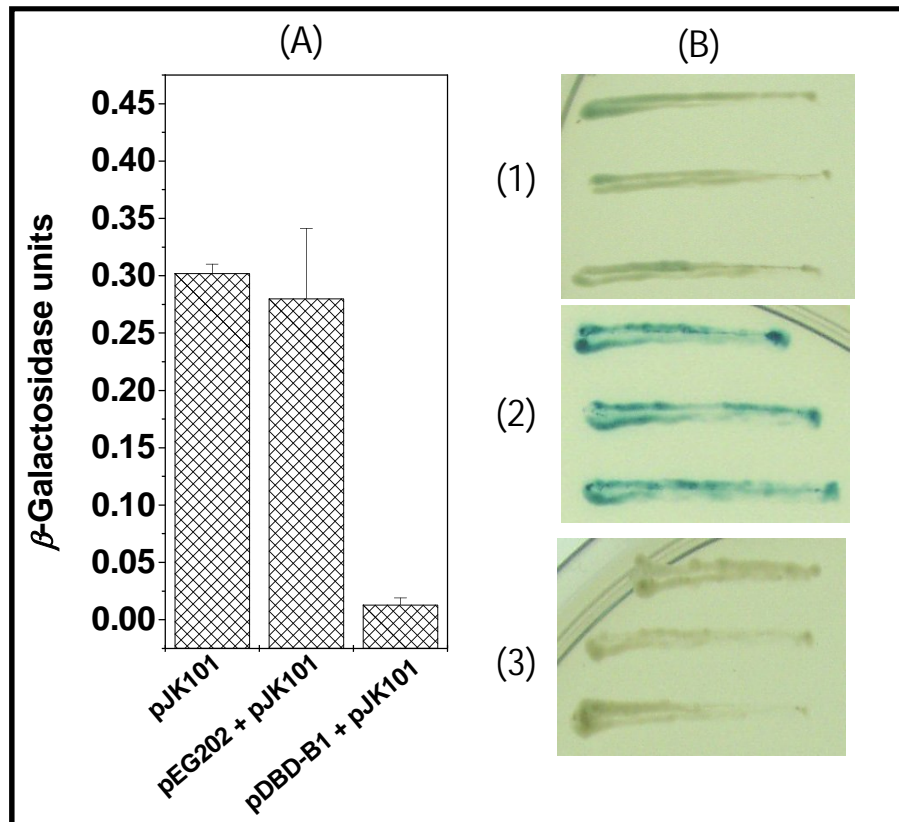

**Figure. 2**

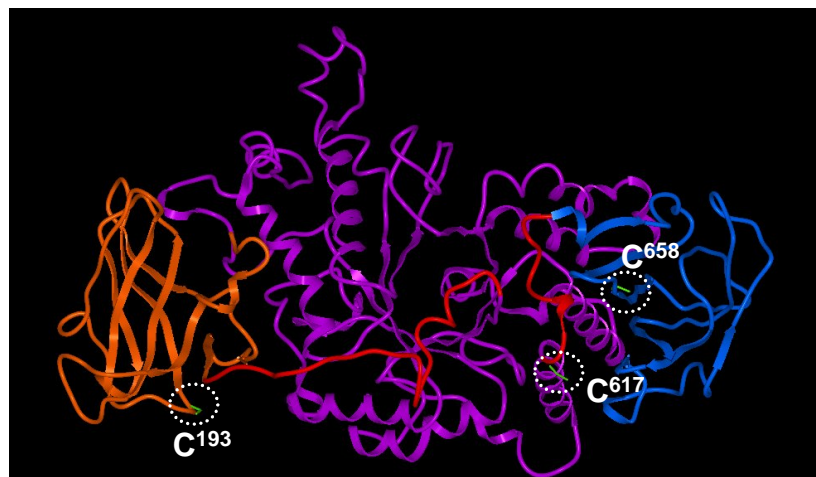

**Figure. 3**

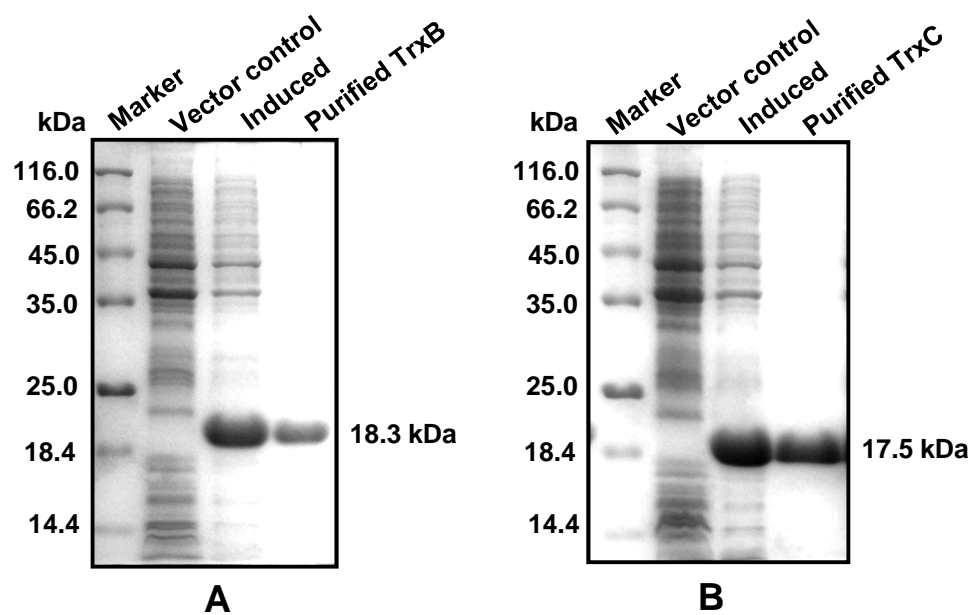

Supplement: Additional file 1 — Figure 1 – Characterization of WhiB1 as a bait. (A) Repression assay to asses the expression and binding of 'bait' to the LexA operator. Four independent transformants of each of the clones were assayed for β-gal expression. Saccharomyces cerevisiae cells (~2 × 10^7^) containing constructs (labeled on the x-axis), were permeabilized with 3 drops of chloroform and 2 drops of 0.1% SDS by vigorous vortexing. Reaction was initiated by adding 0.2 ml ONPG (4 mg/ml) in 'Z buffer' for 3.5 minutes at 28°C. The β-gal activity is expressed as units, on y-axis. (B) WhiB1 lacks intrinsic transcription activity. Saccharomyces cerevisiae cells were transformed with pDBD-B1 (bait), pSH18-34 which contains β-gal as a reporter gene and library plasmid pJG4-5. The β-gal expression was monitored on the Gal+Raff+Ura-X-gal plate. The panels indicate S. cerevisiae containing (1) pSH18-34 + pJG4-5 + pDBD-B1, test (2) pSH18-34 + pJG4-5+ pSH17-4, positive control (3) pSH18-34 + pJG4-5 + pRFHM1, negative control. Figure 2 – Homology model of Mtb GlgB. Three domains are colored differently: N2 domain-orange; TIM-barrel domain-magenta; C-terminal domain-blue. The linker regions between the domains are shown in red. The position of the cysteine residues (C193, C617and C658) on the individual domains is shown as green sticks encircled in dotted boundaries. Figure 3 – Over-expression and purification of TrxB/Rv1471 (1) and TrxC/3914 (2) of Mtb. The proteins were overexpressed in E. coli BL21 (DE3) and were purified by Ni2+-NTA affinity chromatography. Lanes are indicated on the top. [file 1471-2091-10-1-S1.pdf]
