# Supplementary material for: Redox biology of Mycobacterium tuberculosis H37Rv: protein-protein interaction between GlgB and WhiB1 involves exchange of thiol-disulfide
Source: BMC Biochem. 2009 Jan 5;10:1. doi: 10.1186/1471-2091-10-1 (PMC2631452; doi:10.1186/1471-2091-10-1)
Supplement: Additional file 2 — Table 1 – Protein disulfide reductase activity of Mtb TrxB and TrxC. Table 2 – List of primers used in this study [file 1471-2091-10-1-S2.pdf]

Additional file 2

Table 1. Protein disulfide reductase activity of TrxB and TrxC

**Sample**	**Reductase activity ($\Delta A_{650} \times 10^{-3}$ min^{-2})**
TrxB (5μM)	7.20
TrxC (5μM)	6.98
Buffer control	0.57

The reductase activity was measured by insulin disulfide reduction assay as described for WhiB1.

Table 2. List of primers used in this study

**Primer's name**	**Nucleotide sequence**
*whiB*1/WT-Y2H-F	5'-TCC G**GAATTC**ATGGATTGGCGCCAC-3'
*whiB*1/WT-Y2H-R	5'-TTTCG**CTCGAG**GAGT CGTCAGACCC-3'
*whiB*4/WT-Y2H-F	5'-ATATAT**GAATTC**GTGTCAGGAACCCGTCCAGCCG-3'
*whiB*4/WT-Y2H-R	5'-ATATAT**CTCGAG**CTATCCGGCGGTGCCGGTGC-3'
*whiB*1/C40S/F	5′ -TGTAATCGGAGCCCGGTCACCACAG- 3′
*whiB*1/C40S/R	5′ -CTCTGTGGTGACCGGGCTCCGATT-3′
trxB/WT/F	5'-ATATAT**GAATTC**GTGACTACCCGAGACCTCACTGCCGCACAG-3'
trxB/WT/R	5'-ATATAT**CTCGAG**GGCTTGTTGGGCTCGCCCGTTCTGGGTG-3'
trxC/WT/F	5'-ATATAT**GGATCC**ATGACCGATTCCGAGAAGTCCGCCAC-3'
trxC/WT/R	5'-ATATAT**AAGCTT**GTTGAGGTTGGGAACCACGTCTGAGAGCTC-3'

Nucleotide sequences in bold letters are the restriction sites used for cloning, whereas the underlined sequence represent the mutated codon used to replace the cysteine to serine of WhiB1.
